# Phylogeography of the Rock Shell *Thais clavigera* (Mollusca): Evidence for Long-Distance Dispersal in the Northwestern Pacific

**DOI:** 10.1371/journal.pone.0129715

**Published:** 2015-07-14

**Authors:** Xiang Guo, Dan Zhao, Daewui Jung, Qi Li, Ling-Feng Kong, Gang Ni, Tomoyuki Nakano, Akihiko Matsukuma, Sanghee Kim, Chungoo Park, Hyuk Je Lee, Joong-Ki Park

**Affiliations:** 1 Division of EcoScience, Ewha Womans University, 52 Ewhayeodae-gil, Seodaemun-gu, Seoul, 120–750, Republic of Korea; 2 The Key Laboratory of Mariculture, Ministry of Education, Ocean University of China, Qingdao, 266003, China; 3 Program in Cell Biology and Genetics and Department of Parasitology, College of Medicine, Chungbuk National University, Cheongju, 361–763, Republic of Korea; 4 Seto Marine Biological Laboratory, Kyoto University, 459 Nishimuro, Shirahama, Wakayama Prefecture, Japan; 5 Korea Polar Research Institute, 26 Songdomirae-ro Yeonsu-gu, Incheon, 406–840, Republic of Korea; 6 School of Biological Sciences and Technology, Chonnam National University, Gwangju, 500–757, Republic of Korea; 7 Department of Biological Science, College of Science and Engineering, Sangji University, Wonju, 220–702, Republic of Korea; The National Orchid Conservation Center of China; The Orchid Conservation & Research Center of Shenzhen, CHINA

## Abstract

The present-day genetic structure of a species reflects both historical demography and patterns of contemporary gene flow among populations. To precisely understand how these factors shape current population structure of the northwestern (NW) Pacific marine gastropod, *Thais clavigera*, we determined the partial nucleotide sequences of the mitochondrial COI gene for 602 individuals sampled from 29 localities spanning almost the whole distribution of *T*. *clavigera* in the NW Pacific Ocean (~3,700 km). Results from population genetic and demographic analyses (AMOVA, Φ_ST_-statistics, haplotype networks, Tajima’s *D*, Fu’s *F_S_*, mismatch distribution, and Bayesian skyline plots) revealed a lack of genealogical branches or geographical clusters, and a high level of genetic (haplotype) diversity within each of studied population. Nevertheless, low but significant genetic structuring was detected among some geographical populations separated by the Changjiang River, suggesting the presence of geographical barriers to larval dispersal around this region. Several lines of evidence including significant negative Tajima’s *D* and Fu’s *F_S_* statistics values, the unimodally shaped mismatch distribution, and Bayesian skyline plots suggest a population expansion at marine isotope stage 11 (MIS 11; 400 ka), the longest and warmest interglacial interval during the Pleistocene epoch. The lack of genetic structure among the great majority of the NW Pacific *T*. *clavigera* populations may be attributable to high gene flow by current-driven long-distance dispersal of prolonged planktonic larval phase of this species.

## Introduction

The present-day genetic structure of a species reflects both historical demography (i.e., population history) and patterns of contemporary gene flow among populations [1]. An understanding of precisely how these factors shape current population structure has been one of the main issues in ecology and evolutionary biology. Historical demographic events might have a particularly large influence on the genetic structure if a species has not yet reached mutation-drift equilibrium owing to insufficient evolutionary time since a population expansion, which is often the case for marine organisms [2].

A series of marginal seas separates Asia from the Pacific, straddling the world’s largest subduction zone of the Western Pacific [3]. During major glaciations, the large volume of accumulated ice reduced sea levels by approximately 120–140 m [4]. The East China Sea (ECS), the South China Sea (SCS), and the East Sea (ES) (Sea of Japan, SJ) are thought to have become partly continuous landmasses during the glacial period due to the sea level decline in the northwestern (NW) Pacific [5]. Marine organisms in the NW Pacific became extinct or survive only in glacial refuges [6]; however, rising sea levels in the interglacial period resulted in reconnection of these three marginal seas and range extension in marine organisms. Therefore, historical glaciation is one of the important factors that shaped present-day phylogeographical patterns of marine species. Significant genetic differentiation by historical isolation of sea basins in the NW Pacific has been well documented for a variety of marine animal species including crustaceans, molluscs, and fishes [7]. Each marginal sea has been proposed as an independent refuge, resulting in deep phylogeographic divergence among lineages in some marine taxa, such as the red lip mullet *Chelon haematocheilus* [8], the venus clam *Cyclina sinensis* [9], and the mitten crab *Eriocheir sensu stricto* [10]. Compared with records of historical population fragmentation, information on how oceanographic factors influence the genetic structure and distribution of natural populations remains scarce in this region.

**Fig 1:**
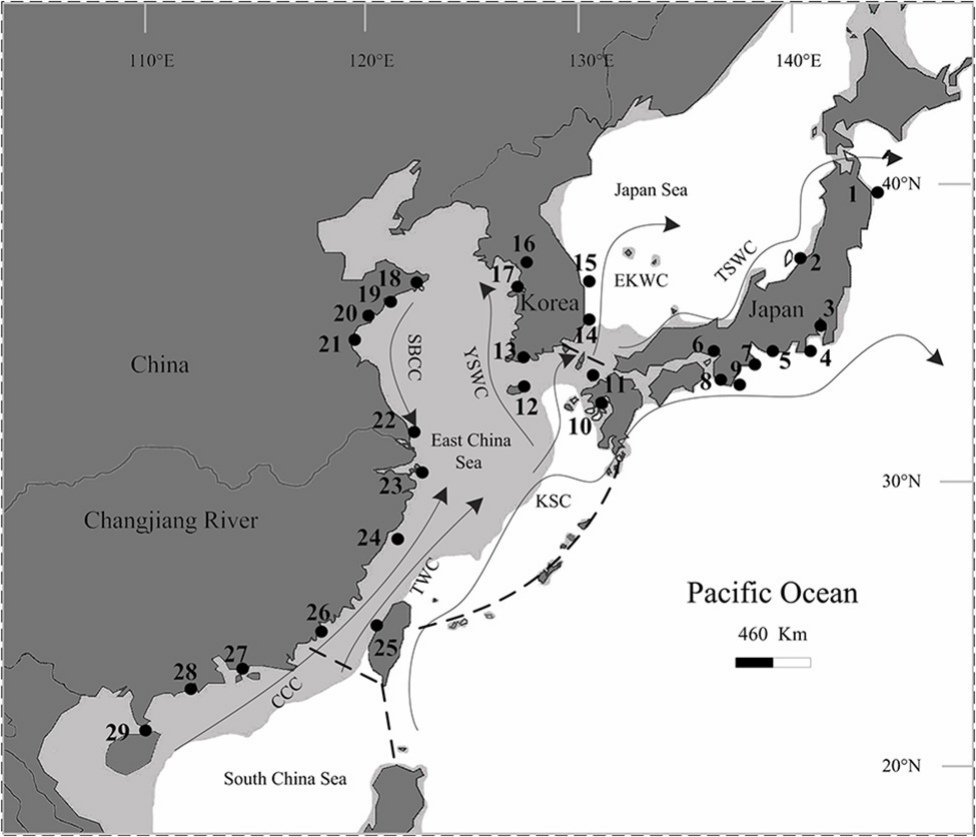
Map of East Asia showing the sampling sites of *Thais clavigera* and the summer ocean currents redrawn from [9]. Populations are labelled with numbers that correspond with those shown in Table 1. Shaded sea areas indicate regions 120 m in depth that would have been exposed during periods of low sea level. EKWC, East Korea Warm Current; TSWC, Tsushima Warm Current; KSC, Kuroshio Current; YSWC, Yellow Sea Warm Current; SBCC, Subei Coastal Current; CCC, China Coastal Current; TWC, Taiwan Warm Current. Dashed lines (——) represent a border of water bodies among South China Sea, East China Sea and Pacific Ocean.

Ocean currents and utilization of a wide range habitat are also correlated with long-distance dispersal, and some marine invertebrates with no geographical structure over large geographic scales have been reported [11–13]. The NW Pacific Sea is characterized by intricate hydrology and geography; two influential ocean current systems (Kuroshio Current [KSC] and China Sea Coastal Current [CSCC]) and freshwater outflows from a continental body (Changjiang River) may play an important role in limiting the dispersal of the planktonic larvae of marine species. Chinese coastlines are influenced by the CSCC, including two currents: the cold Subei Coastal Current (SBCC; flowing southward) and the warm China Coastal Current (CCC; flowing northeastward) (Fig 1). Coastal areas of Japan, western Korea, and eastern Taiwan are influenced by the warm KSC, which includes a strong main stream and three branches, namely, the Tsushima Warm Current (TSWC), Yellow Sea Warm Current (YSWC), and Taiwan Warm Current (TWC). The KSC carries salty water with a relatively high temperature; however, the SBCC carries low salinity surface water along the ECS coast line and the CCC flows into the ECS from the SCS in the summer. Evidence for the influence of different surface water circulation systems on the species distribution and genetic structure of marine organisms has been observed in the acorn barnacle *Tetraclita japonica* in the NW Pacific [14]. It was also proposed that freshwater outflow from the Changjiang River acted as an extrinsic barrier to the southward dispersal of larvae (*Cellana toreuma*) from the north in the NW Pacific [5]. More studies are needed to determine whether the effects of the Changjiang River on genetic structure are species-specific or common in marine species in this area. In general, the three marginal seas (ECS, SCS, and ES [SJ]) in the NW Pacific provide a natural setting to investigate the influences of population fluctuation during Pleistocene oscillations and contemporary gene flow on population structure of marine species.

Most marine invertebrates remain in a pelagic larval stage for several days to months, and they may be able to disperse and metamorphose into sedentary adults [15]. It is generally thought that the propagule duration of marine species is significantly correlated with dispersal capacity [16]. Therefore, marine species with longer planktonic larval stages are expected to exhibit relatively low levels of population structure as a result of their increased opportunity for gene flow [11]. However, some surveys have shown that high levels of gene flow among populations are not always coupled with the duration of the pelagic larval stage. Small, but significant genetic structure has been observed in some marine invertebrates with high dispersal capability, including limpets [17], pen shells [18], and lobsters [19].

The rock shell *Thais clavigera* is the most common gastropod species in intertidal rocky shores of East Asia including China, Korea, Japan, and Taiwan. The life span of this species is a minimum of 7 years [20] and it is capable of adapting to eurythermic and euryhaline environments [21]. Although there is no precise estimate of the pelagic larval duration for *T*. *clavigera*, larvae of its congeneric species *T*. *haemastoma* [22] and *T*. *chocolata* [23] are known to last more than 60 days. Morphologically, *T*. *clavigera* exhibits a wide range of variation in shell sculpture, such as shape, the absence/presence of blotches on the nodules of shell surfaces, and shell apertures [24,25] In a previous DNA barcoding analysis of Korean *Thais* species, a high degree of genetic divergence was discovered in some *T*. *clavigera* populations [26]. In addition, some genetic surveys of *T*. *clavigera* populations performed in certain NW Pacific localities have resulted in inconsistent conclusions [27–29]: Huang [29] revealed significant population structure along the China coast based on the partial sequence of mt COI; however, based on sequence variation in mt16S and COI gene fragments, Wang [27] concluded that there is no genetic differentiation between the ESC and SCS. Moreover, taxon sampling was limited to a few local areas in these studies (Taiwan [28], China Sea [27,29]), insufficient to understand genetic relationships among NW Pacific populations.

In the present study, to better understand contemporary genetic structure and historical demography of the NW Pacific *T*. *clavigera* populations, we sequenced a partial fragment of the mt COI gene from a total of 602 individuals sampled at 29 localities across the NW Pacific Ocean, including both its upstream (Taiwan and China mainland) and downstream (Korea and Japan) ranges. Specifically, we tested whether potential glacial refuges, ocean current systems, or freshwater outflows of the Changjiang River significantly influenced the current population genetic structure of *T*. *clavigera* in the NW Pacific.

## Materials and Methods

### Sample Collection and Sequencing

A total of 602 *T*. *clavigera* specimens were sampled from 29 localities across the NW Pacific Ocean spanning a distance of approximately 3,700 km in East Asia from September 2008 to June 2014 (Fig 1; Table 1). At least 20 individuals were collected and genetically analysed from each locality except two Japanese populations (Iwate and Kanagawa), from which only 10 and 15 samples were obtained, respectively. *T*. *clavigera* is not an endangered or protected species, and therefore all collections were made from public access area without specific permits.

**Table 1:** Sampling information, geographic coordinates, diversity indices, and neutrality tests for 29 *Thais clavigera* populations based on COI.

Locality	Coordinates	N	n	H	*k*	π	Tajima's *D*	Fu's *Fs*
1. Iwate Pref., Japan (IW)	39°38’N, 141°58’E	10	9	0.978	5.05	0.0077	-1.23	**-3.62**
2. Niigate Pref., Japan (NI)	37°55’N, 139°1’E	21	16	0.967	4.67	0.0071	**-1.59**	**-8.35**
3. Kanagawa Pref., Japan (KA)	35°16’N, 139°34’E	15	15	1	6.66	0.0101	**-1.75**	**-10.11**
4. Suzaki, Shizuoka Pref., Japan (SU)	34°37’N, 138°53’E	20	17	0.979	5.47	0.0083	**-1.67**	**-9.84**
5. Aichi Pref., Japan (AI)	34°42’N, 136°58’E	20	18	0.989	6.24	0.0095	**-1.89**	**-11.04**
6. Iwata river, Mie Pref., Japan (MI)	34°31’N, 136°42’E	20	17	0.979	5.26	0.008	**-1.76**	**-10.17**
7. Wakayana Pref., Japan (WA)	34°13’N, 135°9’E	20	17	0.984	5.36	0.0082	**-1.72**	**-10**
8. Shirahama, Wakayama Pref., Japan (SH)	33°41’N, 135°20’E	20	16	0.974	5.11	0.0078	-1.42	**-8.34**
9. Nishinomiya, Hyogo Pref., Japan (HY)	34°43’N, 135°20’E	21	19	0.991	5.65	0.0086	-1.48	**-13.25**
10. Nagasaki Pref., Japan (NA)	32°35’N, 129°45’E	20	16	0.963	5.61	0.0085	**-1.76**	**-7.67**
11. Fukuoka Pref., Japan (FU)	39°38’N, 130°12’E	20	18	0.984	6.26	0.0095	**-1.77**	**-11**
12. Seogwipo-si, Jeju-do, Korea (JJ)	33°13’N, 126°29’E	20	17	0.968	4.91	0.0075	**-2.13**	**-10.76**
13. Wando-gun, Jeollanam-do, Korea (JN)	34°11’N, 126°46’E	20	18	0.99	5.52	0.0084	**-1.97**	**-12.11**
14. Pohang-si, Gyeongsangbuk-do, Korea (PH)	35°59’N, 129°33’E	20	16	0.968	5.38	0.0082	**-1.91**	**-7.96**
15. Uljin-gun, Gyeongsangbuk-do, Korea (UJ)	37°06’N, 129°22’E	20	16	0.963	4.37	0.0066	**-2.13**	**-9.58**
16. Jung-gu, Incheon, Korea (IC)	37°32’N, 126°23’E	20	15	0.963	4.77	0.0072	**-1.96**	**-7.04**
17. Taean-gun, Chungcheongnam-do, Korea (CN)	36°38’N, 126°17’E	20	18	0.99	6.77	0.0102	-1.52	**-10.33**
18. Wendeng, Shandong, China (WD)	36°54´N 122°03´E	30	22	0.97	4.49	0.0068	**-1.77**	**-14.8**
19. Qingdao, Shandong, China (QD)	36°06´N 120°34´E	25	21	0.983	4.8	0.0073	**-1.97**	**-15.62**
20. Rizhao, Shandong, China (RZ)	35°23´N 119°34´E	25	19	0.973	4.23	0.0064	**-1.92**	**-12.49**
21. Lianyungang, Jiangsu, China (LYG)	34°56´N 119°13´E	22	15	0.944	4.65	0.0071	**-1.71**	**-6.19**
22. Nantong, Jiangsu, China (NT)	32°08´N 121°32´E	20	15	0.963	4.38	0.0067	**-1.99**	**-7.63**
23. Zhoushan, Zhejiang, China (ZS)	30°04´N 122°16´E	24	20	0.975	4.88	0.0074	**-1.8**	**-13.96**
24. Wenzhou, Zhejiang, China (WZ)	27°36´N 121°09´E	23	19	0.98	6.36	0.0097	**-1.6**	**-10.06**
25. Changhua, Taiwan (CH)	24°01´N 120°21´E	20	13	0.947	5.8	0.0088	-0.75	-3.17
26. Dongshan, Fujian, China (DS)	23°35´N 117°26´E	22	19	0.983	4.97	0.0076	**-2.02**	**-13.37**
27. Shenzhen, Guangdong, China (SZ)	22°34´N 114°18´E	20	20	1	5.64	0.0086	**-1.98**	**-18.31**
28. Yangjiang, Guangdong, China (YJ)	21°46´N 112°04´E	24	20	0.975	5.38	0.0082	**-1.74**	**-12.96**
29. Haikou, Hainan, China (HK)	20°08´N 110°15´E	20	16	0.974	6.66	0.0101	-0.8	**-6.48**
**Average**		20.8	17	0.976	5.36	0.0081	-1.71	-10.21
**Total**		602	298	0.982	5.34	0.0081	**-2.34**	**-663.21**

Total genomic DNA was extracted using the E.Z.N.A. Mollusc DNA Kit (Omega Bio-Tek Inc., Norcross, GA, USA) following the instructions supplied by the manufacturer. A universal primer set (LCO1490: 5′-GGTCAACAAATCATAAAGATATTGG-3′, HCO2198: 5′-TAAACTTCAGGGTGACCAAAAAATCA-3′) [30] was used to amplify the partial fragment of the mitochondrial COI gene. Polymerase chain reaction (PCR) was performed in a 50 μL reaction volume containing ten units of *Ex-Taq* Polymerase (Takara, Shiga, Japan), 2.5 mM dNTP mixture, 2.5 mM MgCl_2_ and 20 pmole of each primer with the following amplification conditions: an initial 30 s denaturation at 94°C, 40 cycles of 10 s at 98°C, 30 s at 47°C, 30 s at 72°C and a final 10 min extension at 72°C. The sequencing reaction was performed using the BigDye Terminator Cycle Sequencing Kit (Applied Biosystems, Carlsbad, CA, USA) and all COI fragments were sequenced in two directions on an ABI 3730 XL (Applied Biosystems, USA) automatic sequencer.

### Molecular Diversity

Forward and reverse sequences of the COI target fragment were edited, assembled, and merged into consensus sequences using the Geneious software program [31]. Consensus sequences were aligned with ClustalX 1.81 using default settings [32]. Standard molecular diversity indices including haplotype diversity (*h*), nucleotide diversity (*π*), and the mean number of pairwise differences (*k*) were calculated using the Arlequin 3.5 software package [33]. The number of haplotypes was determined by a Bayesian coalescent-based program implemented in DnaSP V5 [34]. All haplotype sequences were deposited in GenBank with the accession numbers KP116312–KP116913.

### Phylogenetic Analyses and Genealogical Network Construction

To infer the phylogenetic relationships among haplotypes, Bayesian inference (BI) and neighbour-joining (NJ) analyses were performed with two congeneric species (*T*. *bronni* and *T*. *luteostoma*) as outgroups using MrBayes 3.1.2 [35] and Mega 6.0 [36], respectively. The Markov-chain Monte Carlo (MCMC) search was run with four chains for 10 million generations with a sampling frequency of 1/1,000 trees. The best-fit model of nucleotide substitutions for Bayesian analyses was estimated using jModeltest v.0.1.1 [37], and selected based on the Akaike Information Criterion ([38]. HKY+G+I was identified as the most appropriate model and used for subsequent analyses. A network showing the genetic relationships among haplotypes was constructed using a median-joining algorithm in Network 4.612 [39]. The maximum parsimony (MP) option was run on the output file to delete all superfluous median vectors and links [40].

### Population Genetic Structure

A hierarchical analysis of molecular variance (AMOVA) was conducted using Arlequin to assess the population genetic structure of *T*. *clavigera* in the NW Pacific. Three independent AMOVA analyses were carried out based on our a priori expectation for each of the focal factors (potential glacial refuges, Changjiang River, and ocean current systems). Our groupings (see Table 2 for details) were as follows (Table 1 & Fig 1 also show sampling sites and location codes, respectively): (1) for glacial refuges, PC (Pacific Ocean) included populations 1 and 3–11, ES (SJ) included 2, 14, and 15, ECS included 12–26, and SCS included 27–29; (2) for Changjiang River, CJN (Changjiang River North) included 18–22 and CJS (Changjiang River South) included 23–24 and 26–29; (3) for ocean systems, KSC included 1–17 and 25, and CSCC included 18–24 and 26–29. These AMOVA analyses partitioned the total molecular variance among groups (Φ_CT_), among populations within groups (Φ_SC_), and among populations whatever the groups (Φ_ST_) and tested if those Φ-statistic values were statistically significant using 10,000 random permutations. The HKY model, which fit the data best according to jModeltest, cannot be implemented in Arlequin, so the Tamura-Nei model was selected to correct for multiple substitutions.

**Table 2:** Analysis of molecular variance (AMOVA) results of population structure. Significant *P*-values are indicated in bold.

Grouping	Source of variation	d.f.	Sum of squares	Variance components	Percentage of variation	Φ-Statistics	*P* value
Potential refugia (PC: 1, 3–11; ES (SJ): 2, 14,15; ECS: 12–26; SCS:27–29)	Among groups	3	27.1	0.04	1.61	Φ_CT_ = 0.016	**< 0.001**
	Among populations within groups	25	85.5	0.04	1.47	Φ_SC_ = 0.015	**< 0.001**
	Within populations	573	1490.9	2.60	96.92	Φ_ST_ = 0.031	**< 0.001**
Changjiang River (CJN: 18–22; CJS: 23–24, 26–29)	Among groups	1	20.5	0.14	5.14	Φ_CT_ = 0.051	**0.003**
	Among populations within groups	9	28.0	0.03	1.02	Φ_SC_ = 0.011	**0.040**
	Within populations	244	608.0	2.49	93.84	Φ_ST_ = 0.062	**< 0.001**
Ocean currents (KSC: 1–17, 25; CCSC: 18–24, 26–29)	Among groups	1	15.4	0.04	1.47	Φ_CT_ = 0.015	**0.002**
	Among populations within groups	27	97.3	0.05	1.80	Φ_SC_ = 0.018	**< 0.001**
	Within populations	573	1490.9	2.60	96.74	Φ_ST_ = 0.033	**< 0.001**

Potential refugia: PC, Pacific Ocean; ECS, East China Sea; SCS, South China Sea; ES, East Sea (SJ, Sea of Japan). Ocean currents: KSC, Kruoshio Current with the branch currents; CCSC, China Coastal Sea Current including China Coastal Current and Subei Coastal Current. Changjiang River: CJS, the South of Changjiang River; CJN, the North of Changjiang River.

Pairwise genetic differentiation between all 29 populations was further assessed with Φ-statistics in Arlequin 3.5 [33]. The significance of each pairwise comparison was estimated using 10,000 permutations, and corrections for multiple tests were made following a sequential Bonferroni procedure [41]. To estimate isolation by distance (IBD), a nonparametric Mantel test was performed online to evaluate the association between matrices of pairwise comparisons among sampling locations of shortest geographical distances (estimated in km using Google Earth version 4.3) and genetic distances (log-transformed) with 10,000 randomizations of the data using Isolation By Distance Web Service 3.23 [42].

### Demographic Analyses

Historical demographic analysis was conducted using two different methods. Tajima’s *D* [43] and Fu’s *Fs* [44] statistics were calculated to test for neutrality using DnaSP. The significance levels of Tajima’s *D* and Fu’s *Fs* were evaluated under 10,000 permutations. The parameter τ obtained from the mismatch distribution was used to estimate the time elapsed since the sudden population expansion using the equation τ = 2μt, where μ is the mutation rate of the marker (per locus per generation) and *t* is the number of generations (Rogers & Harpending, 1992). In order to convert parameters into quantitative estimates of time, we used a mutation rate of 7.9 × 10^−9^ substitutions/site/year, the genus *Nucella*-specific average substitution rate for COI [45], because *Nucella* is ever known the most closely relatives of *T*. *clavigera* (belongs to the same family Muricidae) in which fossil-based molecular clock calibration is thus far available. The generation time was assumed to be 1 year.

For comparison with the mismatch distribution analysis, a Bayesian Skyline Plot (BSP) analysis was executed to examine changes in population size across time in Beast v1.7 software [46]. Three independent MCMC samplings were performed to assure the consistency of the results. Chains were run for 100 million generations and sampled every 1,000 generations, with the first 10% of generations discarded as “burn-in” under the HKY+G+I model determined by jModeltest v.0.1.1 with a constant skyline model and Bayesian skyline tree priors. All operators were optimized automatically. In all runs, the effective sample size yielded by MCMC chains for the parameters of interest was greater than 300. Finally, the results were visualized with the Tracer 1.6 software program [47].

## Results

### Genetic Diversity and Phylogenetic Relationships

A 658 bp sequence of the mt COI gene fragment was determined for 602 individuals of 29 *T*. *clavigera* populations sampled across the NW Pacific Ocean (Table 1; Fig 1). A total of 165 polymorphic sites were identified and 298 haplotypes were encountered. No deletion or insertion mutations were detected. Of these 165 variable sites, 44 were single-nucleotide polymorphic, and the remains were parsimony informative. The COI data in NW Pacific *T*. *clavigera* populations showed a high level of genetic diversity; haplotype diversity (*h*) for all studied populations was 0.982, ranging from 0.944 to 1.000. Nucleotide diversity (*π*) for all examined samples was 0.0081 and ranged from 0.0064 to 0.0102 for individual populations. The average number of nucleotide differences (*k*) between haplotypes was 5.34 (Table 1).

**Fig 2:**
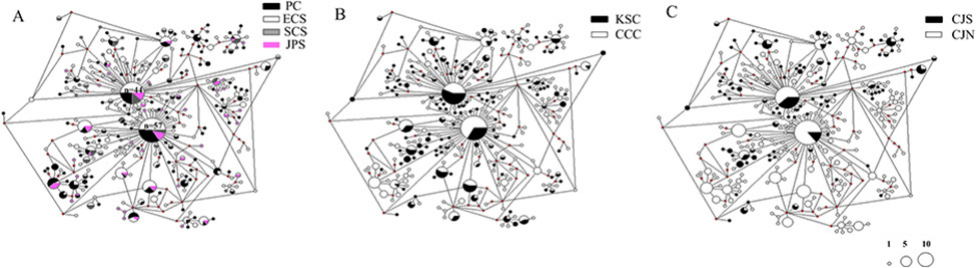
Network of *Thais clavigera* using COI data. The size of circles is proportional to haplotype frequency and median vectors are indicated with a red pie chart. The different shadings of the haplotypes refer to the respective locations in terms of the three population groupings: (a) potential refuges (PC, Pacific Ocean; ECS, East China Sea; SCS, South China Sea; ES, East Sea [SJ, Sea of Japan]); (b) ocean currents (KSC, Kruoshio Current with the branch currents; CCC, China Coastal Current and Subei Coastal Current); (c) Changjiang River (CJS, the South of Changjiang River; CJN, the North of Changjiang River).

The phylogenetic trees for *T*. *clavigera* mtDNA COI sequence data inferred using NJ and BI methods were both unresolved, i.e., all nodes received low bootstrap support (all nodes ≤ 50%), suggesting that the 298 haplotypes sampled in all study populations lacked phylogeographic structure (S1 Fig). For three different groupings based on either potential glacial refuges, the Changjiang River, or the present-day ocean current systems, the centrality of the haplotype networks was occupied by the two most dominant haplotypes; however, these accounted for less than 20% of all sampled individuals (Fig 2). Furthermore, regardless of how we divided these populations into groups, there were no significant genealogical branches or geographic clusters detected in the three haplotype networks. For potential refuges, the two most dominant haplotypes accounted for 16.8% (101/602) of the total individuals (Fig 2A). The most dominant haplotype (9.5%, 57/602) occurred in all populations except Chungcheongnam-do (CN), Pohang-si (PH), Zhoushan (ZS), and Haikou (HK). We detected the second most dominant haplotype (7.3%, 44/602) in 22 out of 29 populations. The great majority of detected haplotypes (76%, 227/298 haplotypes) occurred in only a single individual (i.e., singletons).

### Population Genetic Structure

The three-level hierarchical AMOVA analyses revealed small but statistically significant genetic structure among populations of *T*. *clavigera*, irrespective of whether populations are grouped with respect to potential refuges, Changjiang River, or ocean currents (Table 2). We found that 1.47–5.14% of molecular variation occurred among groups (Φ_CT_; *P* < 0.01), whereas most variation was observed within populations (Φ_ST_; 93.84–96.92% of the total variation, *P* < 0.001). Among the three factors analysed, however, the Changjiang River had the largest influence on genetic structure, indicating it acts as a physical barrier to larval dispersal; the proportion of variation among groups was approximately five times greater than the proportion of variation among populations within groups (Φ_CT_ = 0.051, Φ_SC_ = 0.011; Table 2).

**Fig 3:**
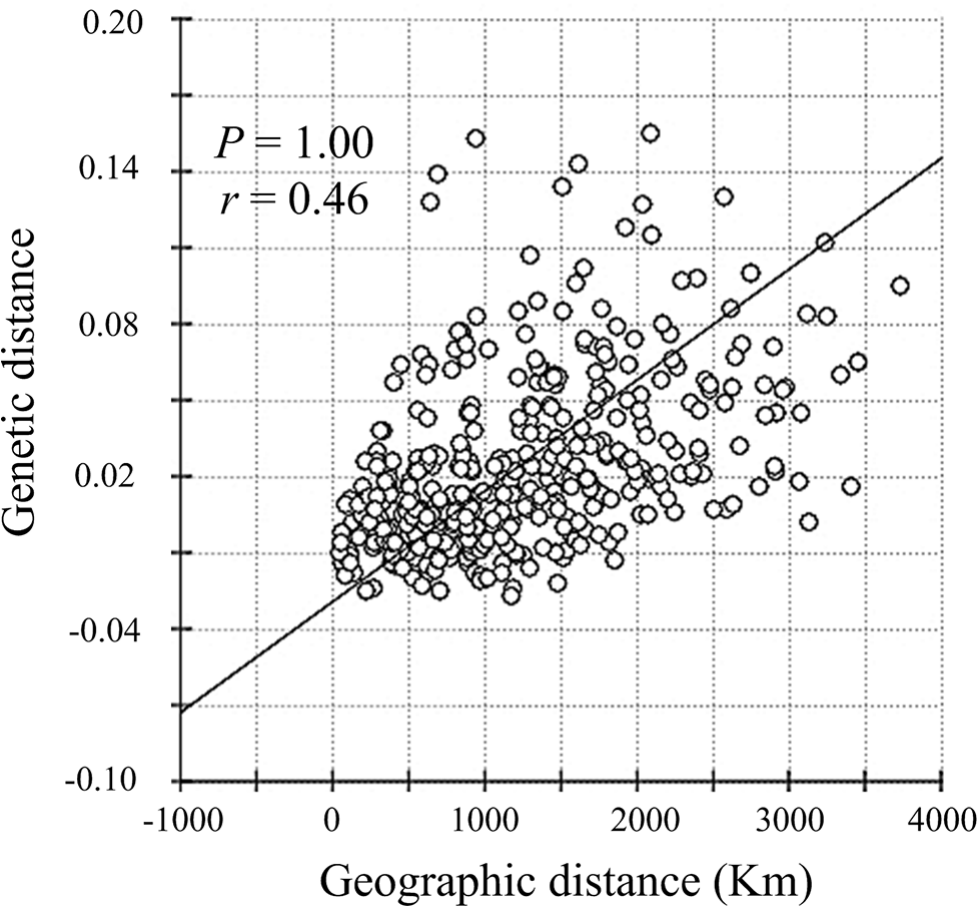
Isolation by distance plot for all *Thais clavigera* populations from the northwestern Pacific Ocean.

Pairwise comparisons across sampling locations showed a near absence of population structure across a wide geographical range (3,700 km). Pairwise Φ_ST_ values between populations varied from -0.027 to 0.155, most of which were non-significant after sequential Bonferroni correction (*P* > 0.05). Interestingly, however, pairwise Φ_ST_ values from eight populations exclusively north or south of the Changjiang River (Suzaki [SU; 4 (location code)], Pohang-si [PH; 14], Qingdao [QD; 19], Rizhao [RZ; 20], Zhoushan [ZS; 23], Wenzhou [WZ; 24], Shenzhen [SZ; 27] and Haikou [HK; 29]) were small, but statistically highly significant (*P* < 0.05) (Table 3). These results substantiate the existence of a barrier to gene flow across the Changjiang River; however, there was no evidence of IBD estimated as a correlation between the shortest geographic distance by sea and genetic distance by the Mantle test (*r* = 0.46, *P* = 1.00) (Fig 3).

**Table 3:** Pairwise Φ_ST_ among *Thais clavigera* populations based on COI (below diagonal) and associated *P*-values (above diagonal) after the sequential Bonferroni correction.

	1	2	3	4	5	6	7	8	9	10	11	12	13	14	15	16	17	18	19	20	21	22	23	24	25	26	27	28	29
	IW	NI	KA	SU	AI	HY	MI	SH	WA	NA	FU	JJ	JN	PH	UJ	IC	CN	WD	QD	RZ	LYG	NT	ZS	WZ	CH	DS	SZ	YJ	HK
1. IW	*	0.091	0.807	0.756	0.366	0.835	0.376	0.387	0.406	0.233	0.663	0.659	0.833	**0.383**	0.255	0.054	0.188	0.038	**0.101**	**0.128**	0.194	0.033	0.001	0.058	0.025	0.041	0.001	0.022	0.01
2. NI	0.038	*	0.421	0.109	0.418	0.068	0.72	0.094	0.433	0.483	0.221	0.265	0.415	**0.002**	0.277	0.297	0.119	0.137	**0**	**0.002**	0.047	0.134	0.025	0.324	0.16	0.29	0.109	0.344	0.204
3. KA	-0.02	0	*	0.687	0.979	0.965	0.488	0.193	0.689	0.688	0.821	0.982	0.984	**0.408**	0.911	0.758	0.71	0.674	**0.231**	**0.598**	0.764	0.243	0.003	0.191	0.078	0.245	0.004	0.126	0.012
4. SU	-0.018	0.022	-0.009	*	0.492	0.905	0.41	0.482	0.457	0.337	0.669	0.826	0.906	**0.782**	0.669	0.125	0.123	0.055	**0.089**	**0.137**	0.034	0.037	0	0.022	0.007	0.046	0.001	0.006	0.002
5. AI	0.004	0	-0.024	-0.002	*	0.877	0.543	0.137	0.36	0.834	0.871	0.748	0.665	**0.336**	0.794	0.717	0.583	0.916	**0.191**	**0.506**	0.585	0.181	0.003	0.165	0.153	0.254	0.006	0.133	0.033
6. HY	-0.025	0.026	-0.025	-0.018	-0.016	*	0.351	0.2	0.293	0.391	0.912	0.934	0.96	**0.613**	0.795	0.383	0.167	0.16	**0.321**	**0.625**	0.437	0.1	0.001	0.039	0.031	0.077	0	0.017	0.002
7. MI	0.003	-0.012	-0.003	-0.001	-0.004	0.003	*	0.433	0.783	0.477	0.289	0.549	0.66	**0.012**	0.274	0.161	0.181	0.164	**0.002**	**0.005**	0.07	0.266	0.013	0.409	0.238	0.294	0.047	0.222	0.114
8. SH	0.001	0.027	0.012	-0.004	0.016	0.011	-0.002	*	0.664	0.215	0.205	0.222	0.275	**0.085**	0.168	0.014	0.014	0.003	**0.001**	**0.002**	0.006	0.023	0	0.035	0.009	0.035	0.001	0.008	0.008
9. WA	-0.001	-0.001	-0.01	-0.003	0.002	0.005	-0.014	-0.011	*	0.263	0.282	0.439	0.658	**0.065**	0.275	0.057	0.043	0.05	**0.001**	**0.005**	0.031	0.085	0.002	0.134	0.08	0.096	0.006	0.039	0.017
10. NA	0.015	-0.004	-0.009	0.004	-0.015	0.002	-0.003	0.013	0.008	*	0.719	0.683	0.441	**0.044**	0.784	0.534	0.397	0.329	**0.041**	**0.15**	0.154	0.167	0.001	0.352	0.135	0.398	0.014	0.173	0.167
11. FU	-0.011	0.011	-0.014	-0.007	-0.015	-0.017	0.005	0.011	0.006	-0.01	*	0.646	0.657	**0.37**	0.857	0.264	0.739	0.411	**0.244**	**0.675**	0.558	0.061	0	0.069	0.075	0.091	0.001	0.029	0.018
12. JJ	-0.012	0.007	-0.024	-0.014	-0.01	-0.019	-0.005	0.01	-0.001	-0.008	-0.006	*	0.962	**0.139**	0.939	0.732	0.083	0.234	**0.046**	**0.188**	0.257	0.269	0.001	0.059	0.031	0.21	0.001	0.054	0.002
13. JN	-0.022	0	-0.027	-0.018	-0.007	-0.021	-0.009	0.006	-0.009	-0.001	-0.006	-0.019	*	**0.31**	0.793	0.438	0.215	0.088	**0.038**	**0.205**	0.162	0.227	0.002	0.104	0.038	0.238	0.002	0.09	0.008
14. PH	0.002	0.066	0.001	-0.012	0.004	-0.007	0.046	0.024	0.027	0.03	0.002	0.016	0.004	*	0.191	0.022	0.054	0.011	**0.379**	**0.575**	0.026	0.001	0	0	0.001	0.002	0	0	0
15. UJ	0.011	0.009	-0.018	-0.009	-0.012	-0.012	0.006	0.015	0.007	-0.015	-0.014	-0.016	-0.011	**0.011**	*	0.608	0.372	0.373	**0.272**	**0.432**	0.416	0.202	0.002	0.117	0.075	0.457	0.001	0.107	0.013
16. IC	0.037	0.006	-0.011	0.015	-0.009	0.001	0.012	0.048	0.026	-0.003	0.006	-0.009	-0.001	**0.038**	-0.006	*	0.227	0.558	**0.082**	**0.303**	0.78	0.566	0.01	0.089	0.059	0.472	0.012	0.196	0.011
17. CN	0.019	0.019	-0.01	0.018	-0.005	0.013	0.013	0.045	0.031	0.001	-0.01	0.019	0.01	**0.026**	0.002	0.009	*	0.458	**0.178**	**0.335**	0.571	0.045	0	0.081	0.081	0.112	0.002	0.1	0.03
18. WD	0.046	0.014	-0.009	0.025	-0.016	0.012	0.013	0.059	0.027	0.003	0.001	0.007	0.018	**0.043**	0.001	-0.005	-0.001	*	**0.043**	**0.193**	0.595	0.065	0.002	0.063	0.045	0.101	0.004	0.04	0.003
19. QD	0.026	0.072	0.009	0.019	0.01	0.004	0.057	0.062	0.063	0.026	0.007	0.024	0.025	**0.002**	0.006	0.022	0.011	0.026	*	**0.902**	0.216	0.003	0	0	0	0.003	0	0	0
20. RZ	0.023	0.056	-0.006	0.013	-0.002	-0.007	0.048	0.06	0.047	0.013	-0.007	0.01	0.009	**-0.005**	0	0.005	0.004	0.01	**-0.014**	*	0.6	0.004	0	0.001	0.002	0.007	0	0.001	0
21.LYG	0.019	0.028	-0.013	0.029	-0.005	-0.001	0.023	0.059	0.035	0.014	-0.005	0.006	0.011	**0.038**	0	-0.013	-0.005	-0.006	**0.009**	**-0.006**	*	0.158	0.004	0.034	0.029	0.206	0.006	0.068	0.002
22. NT	0.052	0.015	0.008	0.035	0.009	0.02	0.007	0.048	0.024	0.013	0.024	0.004	0.007	**0.077**	0.008	-0.006	0.029	0.022	**0.064**	**0.057**	0.013	*	0.177	0.153	0.122	0.984	0.045	0.694	0.019
23. ZS	0.115	0.034	0.061	0.102	0.056	0.085	0.042	0.107	0.066	0.07	0.076	0.068	0.064	**0.153**	0.07	0.045	0.077	0.062	**0.139**	**0.128**	0.06	0.012	*	0.055	0.061	0.149	0.663	0.301	0.032
24. WZ	0.049	0.005	0.014	0.043	0.015	0.033	0	0.043	0.019	0.003	0.027	0.028	0.022	**0.085**	0.024	0.022	0.025	0.024	**0.083**	**0.072**	0.033	0.016	0.024	*	0.529	0.587	0.394	0.519	0.535
25. CH	0.072	0.02	0.03	0.058	0.018	0.041	0.011	0.065	0.03	0.024	0.028	0.038	0.037	**0.096**	0.032	0.032	0.027	0.032	**0.089**	**0.076**	0.043	0.023	0.028	-0.006	*	0.228	0.08	0.328	0.242
26. DS	0.045	0.007	0.007	0.029	0.006	0.021	0.005	0.036	0.02	0.002	0.019	0.007	0.007	**0.068**	-0.002	-0.003	0.019	0.016	**0.057**	**0.047**	0.008	-0.02	0.013	-0.008	0.012	*	0.109	0.98	0.125
27. SZ	0.112	0.022	0.056	0.1	0.049	0.086	0.031	0.098	0.058	0.05	0.074	0.071	0.071	**0.155**	0.08	0.046	0.064	0.054	**0.143**	**0.134**	0.059	0.029	-0.007	0	0.028	0.018	*	0.362	0.249
28. YJ	0.065	0.002	0.018	0.055	0.016	0.044	0.009	0.055	0.032	0.016	0.034	0.026	0.022	**0.097**	0.022	0.011	0.021	0.027	**0.086**	**0.074**	0.024	-0.01	0.004	-0.004	0.004	-0.023	0.002	*	0.271
29. HK	0.095	0.016	0.06	0.083	0.045	0.084	0.024	0.071	0.054	0.021	0.054	0.076	0.063	**0.13**	0.067	0.056	0.046	0.066	**0.127**	**0.118**	0.079	0.052	0.039	-0.008	0.013	0.023	0.01	0.007	*

Significant pairwise Φ_ST_ and *P* values are bolded (*P* < 0.05). See Table 1 for detailed information on site abbreviation.

### Demographic History

**Fig 4:**
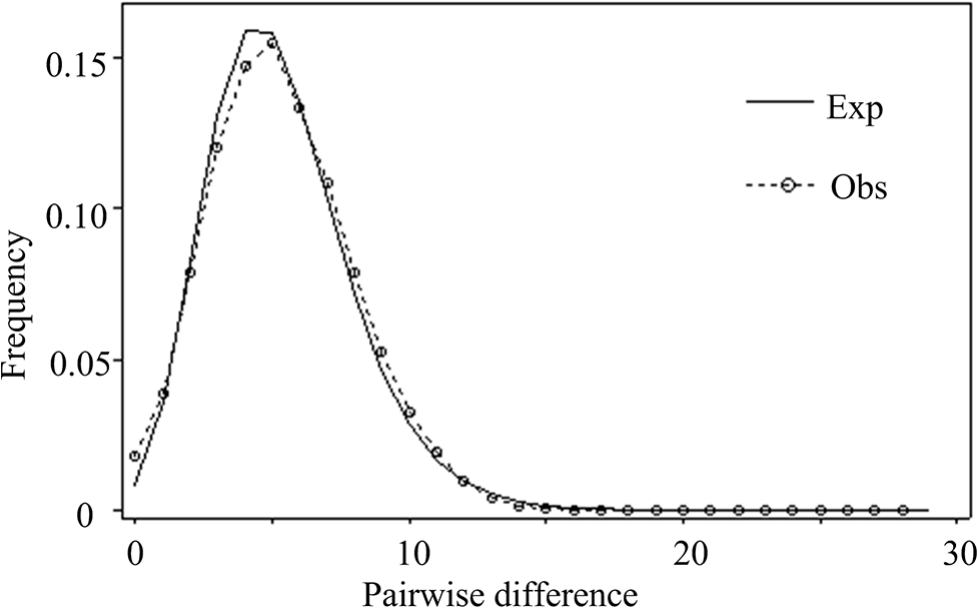
Mismatch distribution of *Thais clavigera* based on COI. The dotted line with circles represents the observed distribution, whereas the solid line shows the expected value under the sudden demographic expansion model.

**Fig 5:**
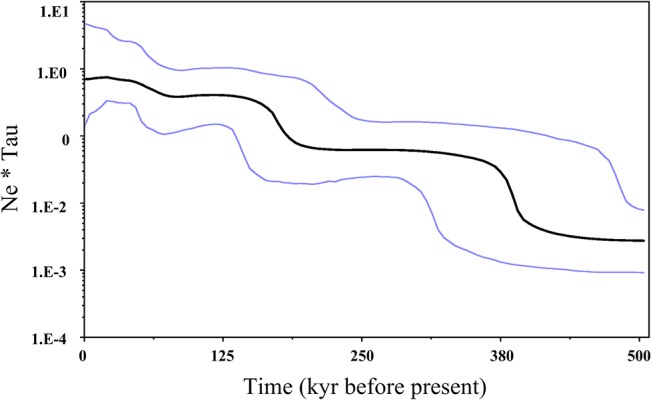
Demographic history of *Thais clavigera* estimated using Bayesian skyline plots from COI data. The black line represents the median population estimates, while the blue lines are the upper and lower bounds of the 95% confidence interval.

We detected significant population expansion of *T*. *clavigera* based on multiple lines of evidence from neutrality tests and the mismatch distribution (Table 1). Tajima’s *D* rejected neutrality (*P <* 0.05) for 23 out of 29 populations. Similarly, Fu’s *Fs* statistic was significantly different from zero (*P <* 0.05) for all populations except for the Taiwan samples. Both tests of neutrality were significant (i.e., indicated significant deviations from neutrality) when samples were grouped as a single data set. The mismatch distribution was unimodal for all examined samples, supporting a model of sudden expansion (Fig 4). The expansion time parameter τ was estimated from mismatch distribution analysis to be 4.005. Assuming a substitution rate of 7.9 × 10^−9^ for COI data, the time since expansion was estimated to be approximately 385 kyr. The population expansion was further validated by the results of BSP analysis, which revealed that population sizes began to expand approximately 400 ka (Fig 5). These estimates indicate a population expansion of *T*. *clavigera* during marine isotope stage (MIS) 11, the longest and warmest interglacial interval of the past 500 kyr.

## Discussion

The genetic structure of a species reflects the effect of historical demography as well as contemporary gene flow among populations. The factors that contribute to the present-day genetic structure can be inferred by genetic analysis of molecular data. In this study, we utilized sequence variation of the partial mtDNA COI gene for 602 individuals sampled from 29 localities spanning an extremely long coastline of approximately 3,700 km. This sampling represents almost the whole distribution of *T*. *clavigera* in the NW Pacific Ocean. We found that genetic variation among the NW Pacific populations was generally low, perhaps owing to a combination of high contemporary gene flow and recent common ancestry of haplotypes. Nevertheless, both AMOVA and pairwise Φ_ST_ analyses indicated weak, but significant genetic structure across the Changjiang River (Tables 2 & 3), suggesting the presence of geographical barriers to continuous larval dispersal at this locality; however, it should be noted that no signal of IBD was detected, nor did the haplotype networks show distinct genealogical branches or geographic clusters (Figs 2 & 3). These results support the hypothesis that the populations of *T*. *clavigera* that were examined have a high level of gene flow throughout the NW Pacific Ocean.

Although there are some exceptions [e.g., the marine clam genus *Lasaea* [48]], marine invertebrate species with a long-lived planktotrophic larval stage are generally capable of long-distance dispersal, and their offspring are spread several hundred to thousand kilometres away from their origin by the prevailing surface flow of the ocean current [16,49,50]; hence, the long-distance dispersal associated with a prolonged pelagic larval stage and the present-day oceanic current have been regarded as the most influential factors contributing to continuous gene flow over a wide geographic scale in many marine invertebrates, including molluscan species [11–13,18]. A reproductive ontogenetic study has reported that *T*. *clavigera* undergoes indirect development with a planktonic veliger larval stage [20], and its pelagic larval stage lasts up to approximately 2 months. Additionally, *T*. *clavigera* is perennial with an average lifespan of 7 years. The species can reach sexual maturity during their second year, and accordingly have a reproductive lifespan of approximately 6 years [20]. It should also be noted that in the NW Pacific, there are two influential current systems in surface water circulation (Fig 1), namely, the KSC (Kuroshio Current) and the CSCC (China Sea Coastal Current), with the KSC flowing northward year-round [51,52], and the CCC (China Coastal Current) entering the ECS (East China Sea) from the SCS (South China Sea) in the summer [7]. These prevailing currents transport a great number of warm-water marine species from their tropical centre to the north and expand their ranges [53]. The long planktonic larval stage in *T*. *clavigera* may facilitates gene flow by current-driven dispersal of pelagic larvae and consequently decreases genetic structure among distant populations spanning over 3,700 km in the NW Pacific coastline. Aside from long-distance dispersal ability and ocean currents, we hypothesize that its utilization of a wide range of habitats is a key factor for successful colonization in a new environments. *T*. *clavigera* is abundant in the intertidal zone over a wide range of environmental conditions including different temperatures and salinities [21]; thus it ability to inhabit a wide range of eurythermic and euryhaline environments may also indicate its potential to colonize new environments.

In this study, the AMOVA analysis and pairwise Φ_ST_ values (Tables 2 & 3) suggest that the Changjiang River poses a weak but significant barrier to gene flow among some *T*. *clavigera* populations, indicating that the larval pool is not well mixed geographically across this area despite the long planktonic larval stage. Nonetheless, it is evident that there is generally a low level of genetic structure among populations, and we did not detect geographic clusters in the haplotype networks, consistent with previous results in marine invertebrates with long-lived pelagic larvae [18,19]. In addition to potential glacial refuges in the NW Pacific region, the Changjiang River and ocean circulation systems are two potentially important geographical barriers shaping current population structure [5,14]. We observed genetic divergence (Φ_CT_ = 0.051, *P* = 0.003) between the northern and southern populations of the Changjiang River in our AMOVA analysis (Table 2). As documented earlier, freshwater outflows from the Changjiang River may act as physical barriers that limit northward dispersal of planktotrophic larvae from southern populations [5]. Genetic subdivision may also be attributed to the habitat of *T*. *clavigera* near the mouth of the Changjiang River. *T*. *clavigera* is most commonly found in shadowy crevices in intertidal rocky shores. There are relatively well developed mudflat areas formed by the deposition of sediments near the mouth of the Changjiang River. These conditions provide a relatively insufficient rocky shore substratum, and are consequentially unsuitable for the settlement of *T*. *clavigera* larvae. When specimens were sampled near the northern mouth of the Changjiang River (e.g., Nantong), only a few *T*. *clavigera* individuals were found; by contrast, near the southern mouth, *T*. *clavigera* were very abundant in the rocky seashore of the Zhoushan archipelago. This habitat discontinuity may have reduced effective gene flow between the northern and southern populations of the Changjiang River.

It has been reported that oceanographic patterns play an important role in maintaining genetic and phenotypic differentiation in the acorn barnacle *Tetraclita japonica* in the NW Pacific [14]. Moreover, in southern Australia, the major ocean currents influence the phylogeography and population structure of the intertidal barnacle *Catomerus polymerus* [54]; however, in the present study of *T*. *clavigera*, we found a lack of genetic structure across major ocean current systems, we found very low, but statistically significant genetic structure between the two major ocean circulation systems (CSCC and KSC) by AMOVA (Φ_CT_ = 0.015, *P* = 0.002), but Φ_SC_ (i.e., structure among populations within groups) was equivalent and statistically significant (Φ_SC_ = 0.018, *P* < 0.001). The lack of genetic structure between the two current systems may be attributable to high gene flow owing to the long spawning time and prolonged planktonic larval phase of *T*. *clavigera*. Our findings that the most common two haplotypes occur at almost every site support this high gene flow hypothesis. Some additional observations suggest that although no water mass from a sub-branch of the KSC reaches the SCS in the summer, in some years this does occur [55]. Moreover, in other seasons, a south-westward current from Kuroshio flowing into the SCS has been observed [55]. In southern China and Taiwan, *T*. *clavigera* spawning could occur from spring to summer (February to August) [20,28]; furthermore, its pelagic larval duration lasts up to approximately 2 months. Therefore, *T*. *clavigera* larvae are likely to enter and mix into the CSCC system from the Taiwan coastline, which may increase gene flow between populations in the two circulation systems to some extent, therefore resulting in a lack of genetic differentiation.

From haplotype network analysis for COI data, we found a complicated network pattern that suggested that *T*. *clavigera* populations underwent a demographic expansion (Fig 2). Also, the observed pattern of mtDNA variation in *T*. *clavigera* further supports the hypothesis of non-equilibrium historical processes such as population range expansion. We observed very high COI haplotype diversity due to an excess of singleton variants (76% of the 298 detected haplotypes) coupled with relatively low nucleotide diversity. The retention of a surplus of rare COI variants may indicate a recent population expansion of *T*. *clavigera*; otherwise, these rare variants are predicted to be eliminated by genetic drift [56]. This suggests that mutation-drift equilibrium has not yet been attained in *T*. *clavigera* in the NW Pacific [57], an interpretation consistent with the significantly negative neutrality test statistics, a clear unimodal mismatch distribution, and BSP analysis. Furthermore, both mismatch distribution and BSP revealed an MIS 11 population expansion approximately 400 ka (Figs 4 & 5). This stage is the longest and the warmest during the Pleistocene epoch [12] and has been described as a super-interglacial period because of its long duration of 25–30 kyr [58]. The sequence of land mollusc species fossils in the Chinese loess-soil shows that the summer monsoon was particularly strengthened during MIS 11, which is typical of warmer climates [59]. Paleontological and palaeoecological estimates of MIS 11 deposits from Japan, Hawaii, Bermuda, and the Bahamas suggest a global sea level had risen during this stage [60–62]. Such climatic conditions are necessary to allow warm-water species to reach the northern Pacific and expand their range.

The Pleistocene glacial age, and particularly the last glacial maximum (LGM) approximately 20,000 years ago, had an important influence on the evolution and genetic structure of marine organisms. Many species in various marine realms appeared to arise at the beginning of LGM [2,63]; however, population expansion of *T*. *clavigera* is assumed to have occurred pre-LGM. These results differ from the traditional view of demographic expansion that it occurs during the period of LGM. Nevertheless, population expansion that occurred pre-LGM has been reported in species such as the cold-water barnacle *Chthamalus challengeri* [13] and the marine snail *Concholepas concholepas* [12]. In the NW Pacific, Ni *et al*. [7] also estimated a period of 120–140 ka that corresponded with dramatic population expansion of various species, including molluscs [13,64], fishes [65,66], and crustaceans [67,68]. These earlier reports indicate the importance of pre-LGM events in determining the demography of marine populations and should be considered in the future. Nonetheless, it is still unclear why the glacial events did not significantly impact the current population structure and demographic history of these species.

## Conclusions

To better understand contemporary genetic structure and historical demography of the NW Pacific *T*. *clavigera* populations, we determined the partial sequence of the mt COI gene from 602 individuals sampled from 29 localities across the NW Pacific Ocean. We observed a high level of genetic diversity within each of sampled populations, and no significant genealogical branches or geographic clusters, suggesting high levels of gene flow among populations throughout the NW Pacific. Nevertheless, we detected low, but significant genetic differentiation that corresponds to habitat conditions and freshwater discharge from the Changjiang River. Since we used only a single mtDNA marker, further studies of *T*. *clavigera* using multiple nuclear markers are required to validate the observed genetic structure. Also, population genetic studies of other marine species with a planktonic larval phase in the region will provide additional insight into the phylogeographic patterns of NW Pacific organisms.

## Supporting Information

S1 Fig Neighbour-joining (NJ) tree of COI haplotypes for populations of *Thais clavigera* from the northwestern Pacific. Both NJ and Bayesian inference (BI) analyses yield the same topology. Bootstrap values for NJ (the former number) and the posterior probabilities for BI (the latter number) analyses are indicated at the nodes.
(TIF)
