# Correction: Phylogeography of the Rock Shell Thais clavigera (Mollusca): Evidence for Long-Distance Dispersal in the Northwestern Pacific

**DOI:** 10.1371/journal.pone.0135540

**Published:** 2015-08-07

**Authors:** Xiang Guo, Dan Zhao, Daewui Jung, Qi Li, Ling-Feng Kong, Gang Ni, Tomoyuki Nakano, Akihiko Matsukuma, Sanghee Kim, Chungoo Park, Hyuk Je Lee, Joong-Ki Park

Fig 1 is incorrectly labeled. Please view Fig 1 here.

**Fig 1:**
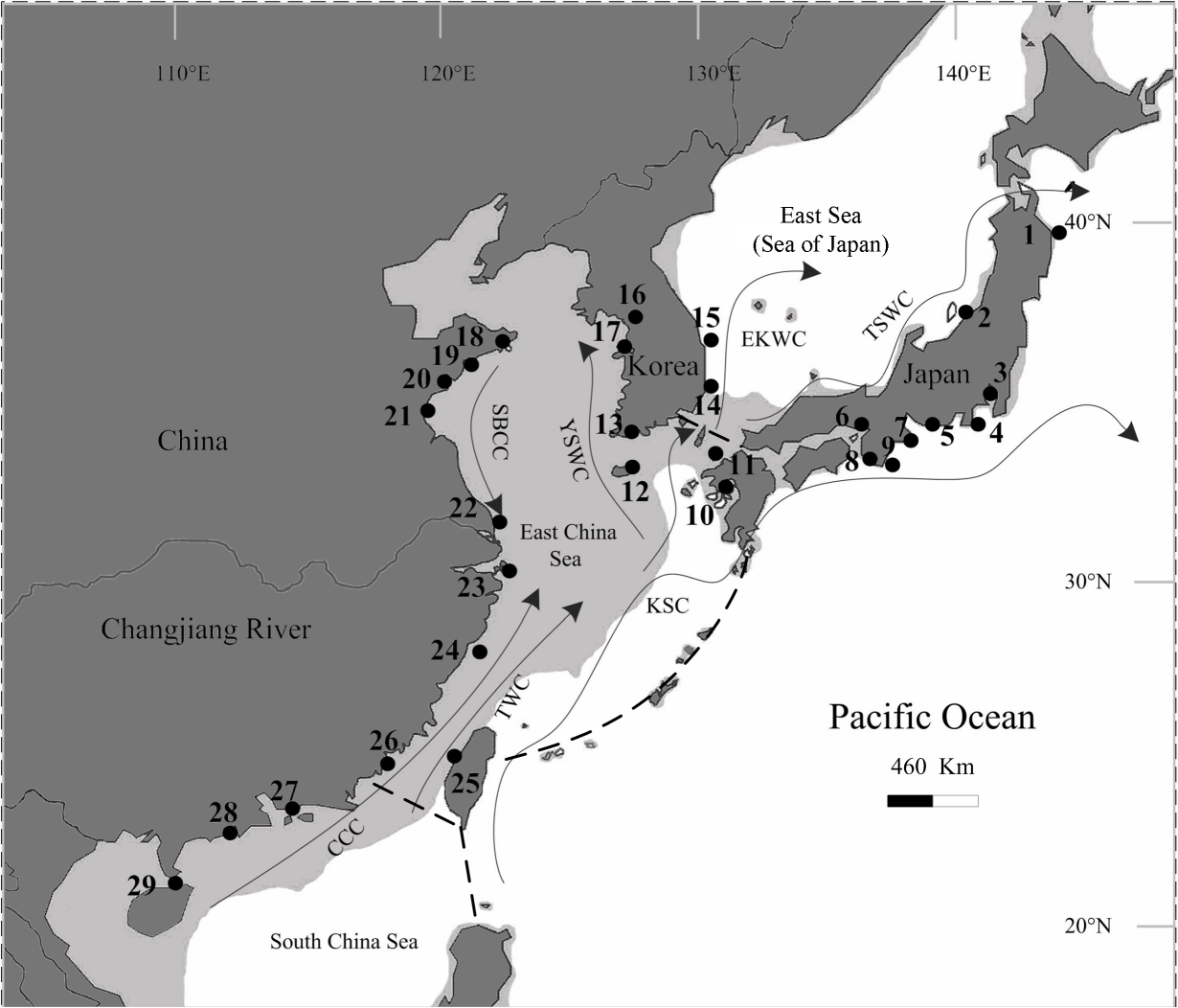
Map of East Asia showing the sampling sites of *Thais clavigera* and the summer ocean currents redrawn from [9]. Populations are labelled with numbers that correspond with those shown in Table 1. Shaded sea areas indicate regions 120 m in depth that would have been exposed during periods of low sea level. EKWC, East Korea Warm Current; TSWC, Tsushima Warm Current; KSC, Kuroshio Current; YSWC, Yellow Sea Warm Current; SBCC, Subei Coastal Current; CCC, China Coastal Current; TWC, Taiwan Warm Current. Dashed lines (——) represent a border of water bodies among South China Sea, East China Sea and Pacific Ocean.
