# Supplementary figures and images for: Phylogeography of the Rock Shell Thais clavigera (Mollusca): Evidence for Long-Distance Dispersal in the Northwestern Pacific
Source: PLoS One. 2015 Jul 14;10(7):e0129715. doi: 10.1371/journal.pone.0129715 (PMC4501670; doi:10.1371/journal.pone.0129715)

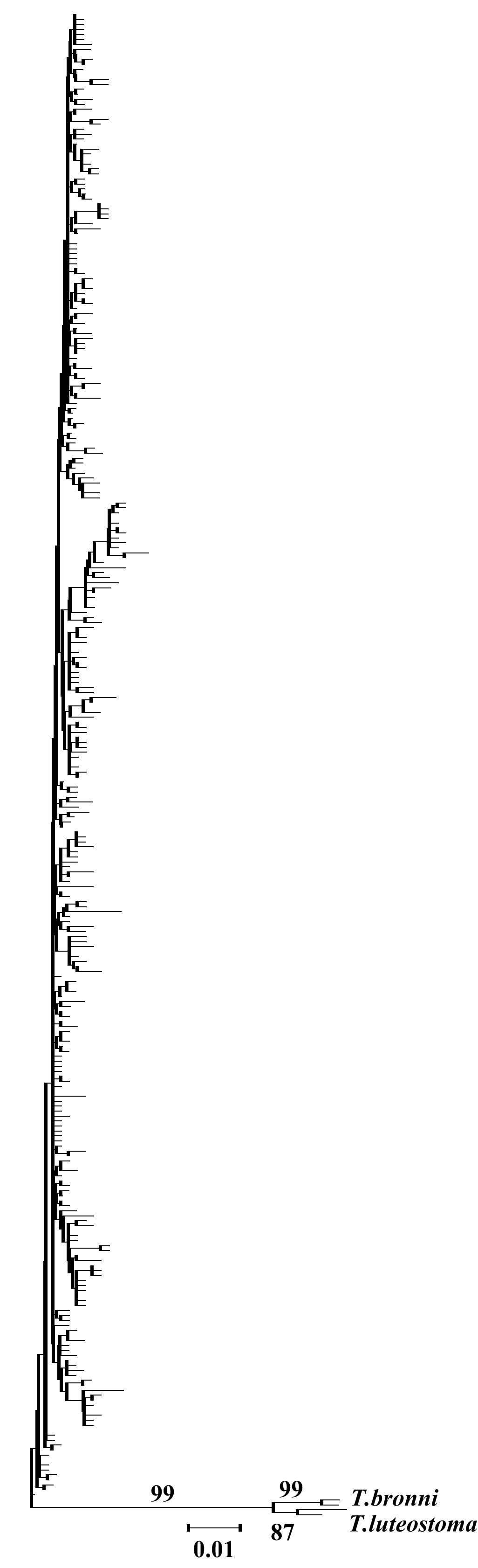

Supplement: S1 Fig — Both NJ and Bayesian inference (BI) analyses yield the same topology. Bootstrap values for NJ (the former number) and the posterior probabilities for BI (the latter number) analyses are indicated at the nodes. (TIF) [file pone.0129715.s001.tif]
